# 3-Cyano-*N*-methylpyridinium perchlorate

**DOI:** 10.1107/S1600536814014421

**Published:** 2014-06-25

**Authors:** Cameron A. McCormick, Vu D. Nguyen, Lynn V. Koplitz, Joel T. Mague

**Affiliations:** a Department of Physics, Loyola University, New Orleans, LA 70118, USA; b Department of Chemistry, Loyola University, New Orleans, LA 70118, USA; c Department of Chemistry, Tulane University, New Orleans, LA 70118, USA

**Keywords:** crystal structure

## Abstract

In the crystal of the title mol­ecular salt, C_7_H_7_N_2_^+^·ClO_4_^−^, the components are linked by C—H⋯O and C—H⋯N inter­actions, generating zigzag chains running parallel to [100].

## Related literature   

For structures of other 3-cyano-1-methyl­pyridinium salts, see: Koplitz *et al.* (2003 ▶); Mague *et al.* (2005 ▶); Zhu *et al.* (1999 ▶). For the structure of 4-cyano-1-methyl­pyridinium perchlorate, see: Nguyen *et al.* (2014 ▶). For a discussion of anion–π inter­actions, see: Frontera *et al.* (2011 ▶). In contrast to the structure found for the title compound, the structures of the isomeric salts 2-cyano-1-methyl­pyridinium nitrate (Koplitz *et al.*, 2003 ▶) and 2-cyano­anilinium nitrate (Cui & Wen, 2008 ▶) crystallize in flat layers of two-dimensional networks with only a few atoms protruding from the mirror plane while 3-cyano­anilinium nitrate (Wang, 2009 ▶) forms a more open structure.
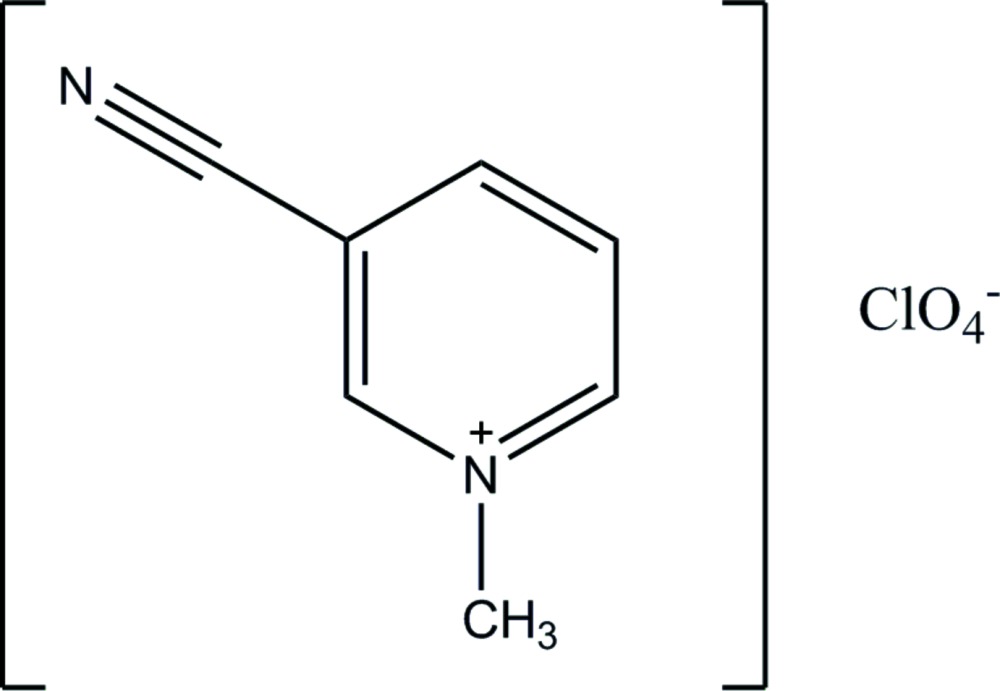


## Experimental   

### 

#### Crystal data  

C_7_H_7_N_2_^+^·ClO_4_^−^
*M* *_r_* = 218.60
Monoclinic, 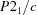

*a* = 8.1490 (7) Å
*b* = 7.7338 (7) Å
*c* = 14.5297 (13) Å
β = 97.522 (1)°
*V* = 907.82 (14) Å^3^
*Z* = 4
Mo *K*α radiation
μ = 0.41 mm^−1^
*T* = 120 K
0.26 × 0.24 × 0.05 mm

#### Data collection  

Bruker SMART APEX CCD diffractometer
Absorption correction: multi-scan (*SADABS*; Bruker, 2010 ▶) *T*_min_ = 0.89, *T*_max_ = 0.98
15448 measured reflections
2364 independent reflections
2187 reflections with *I* > 2σ(*I*)
*R*_int_ = 0.031

#### Refinement  

*R*[*F* ^2^ > 2σ(*F* ^2^)] = 0.032
*wR*(*F* ^2^) = 0.089
*S* = 1.10
2364 reflections
128 parameters
H-atom parameters constrained
Δρ_max_ = 0.33 e Å^−3^
Δρ_min_ = −0.42 e Å^−3^

### 

Data collection: *APEX2* (Bruker, 2010 ▶); cell refinement: *SAINT* (Bruker, 2010 ▶); data reduction: *SAINT*; program(s) used to solve structure: *SHELXS97* (Sheldrick, 2008 ▶); program(s) used to refine structure: *SHELXL97* (Sheldrick, 2008 ▶); molecular graphics: *DIAMOND* (Brandenburg & Putz, 2012 ▶); software used to prepare material for publication: *SHELXTL* (Sheldrick, 2008 ▶).

## Supplementary Material

Crystal structure: contains datablock(s) I, global. DOI: 10.1107/S1600536814014421/hb7237sup1.cif

Structure factors: contains datablock(s) I. DOI: 10.1107/S1600536814014421/hb7237Isup2.hkl

Supporting information file. DOI: 10.1107/S1600536814014421/hb7237Isup3.cml

CCDC reference: 1009069

Additional supporting information:  crystallographic information; 3D view; checkCIF report

## Figures and Tables

**Table 1:** Hydrogen-bond geometry (Å, °)

*D*—H⋯*A*	*D*—H	H⋯*A*	*D*⋯*A*	*D*—H⋯*A*
C1—H1*A*⋯O2^i^	0.98	2.56	3.5377 (19)	173
C1—H1*A*⋯O3^i^	0.98	2.59	3.1868 (17)	119
C1—H1*B*⋯N2^ii^	0.98	2.56	3.3136 (19)	134
C1—H1*B*⋯O2	0.98	2.62	3.3759 (17)	134
C2—H2⋯O1^iii^	0.95	2.22	3.1577 (16)	168

Symmetry codes: (i) 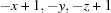; (ii) 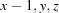; (iii) 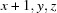.

**Fig. 1.**
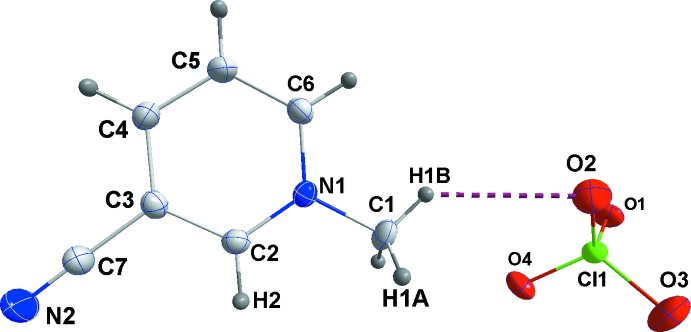
Perspective view of I with displacement ellipsoids drawn at the 50% probability level and H-atoms as spheres of arbitrary radius.

**Fig. 2.**
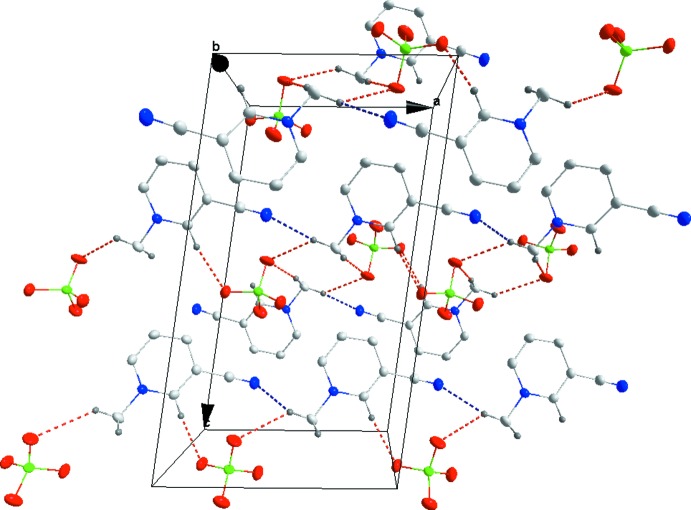
Packing projected down the b axis with C—H···O interactions shown as red dotted lines and C—H···N interactions as blue dotted lines.

**Fig. 3.**
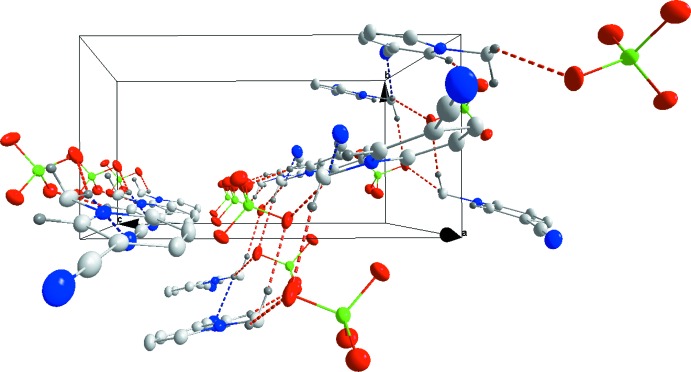
Packing projected onto (100) with C—H···O interactions shown as red dotted lines and C—H···N interactions as blue dotted lines.

## supplementary crystallographic information

### S1. Comment

A perspective view of the title compound appears in Fig. 1 while Fig. 2 illustrates the zigzag rows of anions with cations bound on either side *via* C—H···O hydrogen bonds (Table 1). Additionally, there are C—H···N interactions between methyl H atoms of one cation and the cyano group of the next cation in the chain. An end view of these motifs is shown in Fig. 3. A notable feature is the close cation-anion contact (H1A···O2^i^ = 2.56 Å (symmetry code: (i) 1 - *x*, *-y*, 1 - *z*) which is strikingly similar to the motif that dominates the structure of 2-cyano-1-methylpyridinium nitrate (Koplitz *et al.*, 2003). These close contacts are likely the result of electrostatic cation-anion attraction with the orientation possibly reinforced by an anion-π interaction (Frontera *et al.*, 2011). In contrast to the structure found for the title compound, the structures of the isomeric salts 2-cyano-1-methylpyridinium nitrate (Koplitz *et al.*, 2003) and 2-cyanoanilinium nitrate (Cui & Wen, 2008) crystallize in flat layers of two-dimensional networks with only a few atoms protruding from the mirror plane while 3-cyanoanilinium nitrate (Wang, 2009) forms a more open structure.

### S2. Experimental

3-Cyanopyridine (10.55 g) was dissolved in benzene (40 ml). Iodomethane (9.5 ml) was added to this solution slowly with stirring and the solution was refluxed for 75 minutes. Yellow solid 3-cyano-1-methylpyridinium iodide (m.p. 196° C, blood-red melt) was collected by vacuum filtration. This solid (0.98 g) was then dissolved in a solution of silver perchlorate previously prepared by reacting Ag_2_O (0.47 g) with 0.5 *M* aqueous HClO_4_(8.0 ml). After stirring, precipitated AgI was removed by vacuum filtration and the filtrate containing 3-cyano-1-methylpyridinium perchlorate was slowly evaporated to dryness to form colorless plates of the title compound.

### S3. Refinement

H-atoms were placed in calculated positions (C—H = 0.95 - 0.98 Å) and included as riding contributions with isotropic displacement parameters 1.2 - 1.5 times those of the attached carbon atoms.

### Crystal data

**Table:**

C_7_H_7_N_2_^+^·ClO_4_^−^	*F*(000) = 448
*M**_r_* = 218.60	*D*_x_ = 1.599 Mg m^−^^3^
Monoclinic, *P*2_1_/*c*	Mo *K*α radiation, λ = 0.71073 Å
*a* = 8.1490 (7) Å	Cell parameters from 9885 reflections
*b* = 7.7338 (7) Å	θ = 2.5–29.1°
*c* = 14.5297 (13) Å	µ = 0.41 mm^−^^1^
β = 97.522 (1)°	*T* = 120 K
*V* = 907.82 (14) Å^3^	Plate, colourless
*Z* = 4	0.26 × 0.24 × 0.05 mm

### Data collection

**Table:**

Bruker SMART APEX CCD diffractometer	2364 independent reflections
Radiation source: fine-focus sealed tube	2187 reflections with *I* > 2σ(*I*)
Graphite monochromator	*R*_int_ = 0.031
φ and ω scans	θ_max_ = 29.1°, θ_min_ = 2.5°
Absorption correction: multi-scan (*SADABS*; Bruker, 2010)	*h* = −11→11
*T*_min_ = 0.89, *T*_max_ = 0.98	*k* = −10→10
15448 measured reflections	*l* = −19→19

### Refinement

**Table:**

Refinement on *F*^2^	Primary atom site location: structure-invariant direct methods
Least-squares matrix: full	Secondary atom site location: difference Fourier map
*R*[*F*^2^ > 2σ(*F*^2^)] = 0.032	Hydrogen site location: inferred from neighbouring sites
*wR*(*F*^2^) = 0.089	H-atom parameters constrained
*S* = 1.10	*w* = 1/[σ^2^(*F*_o_^2^) + (0.0451*P*)^2^ + 0.4231*P*] where *P* = (*F*_o_^2^ + 2*F*_c_^2^)/3
2364 reflections	(Δ/σ)_max_ = 0.001
128 parameters	Δρ_max_ = 0.33 e Å^−^^3^
0 restraints	Δρ_min_ = −0.42 e Å^−^^3^

### Special details

**Table:**

Experimental. The diffraction data were obtained from 3 sets of 400 frames, each of width 0.5 °. in omega, colllected at phi = 0.00, 90.00 and 180.00 °. and 2 sets of 800 frames, each of width 0.45 ° in phi, collected at omega = -30.00 and 210.00 °. The scan time was 15 sec/frame.
Geometry. All e.s.d.'s (except the e.s.d. in the dihedral angle between two l.s. planes) are estimated using the full covariance matrix. The cell e.s.d.'s are taken into account individually in the estimation of e.s.d.'s in distances, angles and torsion angles; correlations between e.s.d.'s in cell parameters are only used when they are defined by crystal symmetry. An approximate (isotropic) treatment of cell e.s.d.'s is used for estimating e.s.d.'s involving l.s. planes.
Refinement. Refinement of *F*^2^ against ALL reflections. The weighted *R*-factor *wR* and goodness of fit *S* are based on *F*^2^, conventional *R*-factors *R* are based on *F*, with *F* set to zero for negative *F*^2^. The threshold expression of *F*^2^ > σ(*F*^2^) is used only for calculating *R*-factors(gt) *etc*. and is not relevant to the choice of reflections for refinement. *R*-factors based on *F*^2^ are statistically about twice as large as those based on *F*, and *R*- factors based on ALL data will be even larger. H-atoms were placed in calculated positions (C—H = 0.95 - 0.98 Å) and included as riding contributions with isotropic displacement parameters 1.2 - 1.5 times those of the attached carbon atoms.

### Fractional atomic coordinates and isotropic or equivalent isotropic displacement parameters (Å^2^)

**Table:**

	*x*	*y*	*z*	*U*_iso_*/*U*_eq_	
N1	0.67687 (13)	0.33669 (13)	0.36093 (7)	0.0178 (2)	
N2	1.23923 (15)	0.4848 (2)	0.35273 (9)	0.0343 (3)	
C1	0.58669 (16)	0.28809 (18)	0.43927 (9)	0.0243 (3)	
H1A	0.6309	0.1789	0.4663	0.036*	
H1B	0.4687	0.2743	0.4167	0.036*	
H1C	0.6008	0.3790	0.4867	0.036*	
C2	0.84053 (15)	0.36279 (16)	0.37864 (9)	0.0197 (2)	
H2	0.8961	0.3454	0.4396	0.024*	
C3	0.92864 (15)	0.41495 (16)	0.30835 (9)	0.0193 (2)	
C4	0.84807 (16)	0.43730 (17)	0.21847 (9)	0.0215 (3)	
H4	0.9072	0.4724	0.1695	0.026*	
C5	0.67914 (16)	0.40673 (19)	0.20269 (9)	0.0240 (3)	
H5	0.6210	0.4197	0.1420	0.029*	
C6	0.59519 (15)	0.35749 (17)	0.27498 (9)	0.0212 (2)	
H6	0.4791	0.3382	0.2640	0.025*	
C7	1.10263 (16)	0.45190 (19)	0.33204 (9)	0.0246 (3)	
Cl1	0.21118 (3)	0.17038 (4)	0.56832 (2)	0.01789 (10)	
O1	0.05612 (12)	0.26105 (14)	0.56747 (7)	0.0284 (2)	
O2	0.22598 (14)	0.10417 (15)	0.47740 (7)	0.0338 (3)	
O3	0.21765 (14)	0.02981 (14)	0.63375 (7)	0.0342 (3)	
O4	0.34636 (12)	0.28745 (14)	0.59632 (8)	0.0298 (2)	

### Atomic displacement parameters (Å^2^)

**Table:**

	*U*^11^	*U*^22^	*U*^33^	*U*^12^	*U*^13^	*U*^23^
N1	0.0195 (5)	0.0164 (5)	0.0182 (5)	0.0014 (4)	0.0051 (4)	0.0010 (4)
N2	0.0211 (6)	0.0475 (8)	0.0342 (7)	0.0006 (5)	0.0028 (5)	0.0047 (6)
C1	0.0247 (6)	0.0263 (6)	0.0239 (6)	0.0022 (5)	0.0109 (5)	0.0051 (5)
C2	0.0198 (6)	0.0207 (6)	0.0182 (5)	0.0024 (4)	0.0008 (4)	0.0013 (4)
C3	0.0170 (5)	0.0191 (5)	0.0218 (6)	0.0022 (4)	0.0029 (4)	0.0007 (4)
C4	0.0216 (6)	0.0257 (6)	0.0180 (5)	0.0014 (5)	0.0058 (4)	0.0006 (5)
C5	0.0223 (6)	0.0323 (7)	0.0170 (5)	0.0007 (5)	0.0006 (4)	−0.0006 (5)
C6	0.0174 (5)	0.0243 (6)	0.0215 (6)	−0.0008 (4)	0.0013 (4)	−0.0024 (5)
C7	0.0205 (6)	0.0301 (7)	0.0235 (6)	0.0023 (5)	0.0038 (5)	0.0032 (5)
Cl1	0.01977 (16)	0.01840 (16)	0.01568 (16)	0.00044 (9)	0.00301 (10)	−0.00022 (9)
O1	0.0199 (4)	0.0324 (5)	0.0315 (5)	0.0058 (4)	−0.0013 (4)	0.0000 (4)
O2	0.0450 (6)	0.0383 (6)	0.0199 (5)	−0.0080 (5)	0.0118 (4)	−0.0091 (4)
O3	0.0465 (6)	0.0273 (5)	0.0317 (5)	0.0102 (5)	0.0160 (5)	0.0121 (4)
O4	0.0216 (5)	0.0303 (5)	0.0359 (6)	−0.0040 (4)	−0.0018 (4)	−0.0065 (4)

### Geometric parameters (Å, º)

**Table:**

N1—C2	1.3401 (16)	C3—C7	1.4431 (17)
N1—C6	1.3457 (16)	C4—C5	1.3860 (18)
N1—C1	1.4819 (16)	C4—H4	0.9500
N2—C7	1.1430 (18)	C5—C6	1.3799 (18)
C1—H1A	0.9800	C5—H5	0.9500
C1—H1B	0.9800	C6—H6	0.9500
C1—H1C	0.9800	Cl1—O2	1.4365 (10)
C2—C3	1.3832 (17)	Cl1—O3	1.4406 (10)
C2—H2	0.9500	Cl1—O4	1.4427 (10)
C3—C4	1.3935 (17)	Cl1—O1	1.4437 (10)
			
C2—N1—C6	121.27 (11)	C5—C4—H4	121.0
C2—N1—C1	118.15 (11)	C3—C4—H4	121.0
C6—N1—C1	120.56 (11)	C6—C5—C4	120.12 (12)
N1—C1—H1A	109.5	C6—C5—H5	119.9
N1—C1—H1B	109.5	C4—C5—H5	119.9
H1A—C1—H1B	109.5	N1—C6—C5	120.34 (12)
N1—C1—H1C	109.5	N1—C6—H6	119.8
H1A—C1—H1C	109.5	C5—C6—H6	119.8
H1B—C1—H1C	109.5	N2—C7—C3	177.92 (16)
N1—C2—C3	120.12 (11)	O2—Cl1—O3	109.73 (7)
N1—C2—H2	119.9	O2—Cl1—O4	109.28 (6)
C3—C2—H2	119.9	O3—Cl1—O4	109.08 (7)
C2—C3—C4	120.11 (11)	O2—Cl1—O1	110.12 (7)
C2—C3—C7	118.06 (11)	O3—Cl1—O1	109.18 (6)
C4—C3—C7	121.78 (11)	O4—Cl1—O1	109.44 (6)
C5—C4—C3	118.02 (11)		
			
C6—N1—C2—C3	1.11 (18)	C7—C3—C4—C5	−176.95 (13)
C1—N1—C2—C3	−177.46 (11)	C3—C4—C5—C6	0.7 (2)
N1—C2—C3—C4	−1.25 (19)	C2—N1—C6—C5	−0.06 (19)
N1—C2—C3—C7	176.16 (12)	C1—N1—C6—C5	178.47 (12)
C2—C3—C4—C5	0.36 (19)	C4—C5—C6—N1	−0.8 (2)

### Hydrogen-bond geometry (Å, º)

**Table:**

*D*—H···*A*	*D*—H	H···*A*	*D*···*A*	*D*—H···*A*
C1—H1*A*···O2^i^	0.98	2.56	3.5377 (19)	173
C1—H1*A*···O3^i^	0.98	2.59	3.1868 (17)	119
C1—H1*B*···N2^ii^	0.98	2.56	3.3136 (19)	134
C1—H1*B*···O2	0.98	2.62	3.3759 (17)	134
C2—H2···O1^iii^	0.95	2.22	3.1577 (16)	168

Symmetry codes: (i) −*x*+1, −*y*, −*z*+1; (ii) *x*−1, *y*, *z*; (iii) *x*+1, *y*, *z*.
